# Employment transitions for spouses of stroke survivors: evidence from Swedish national registries

**DOI:** 10.1186/s12889-020-09625-1

**Published:** 2020-10-07

**Authors:** Josefine Persson, Gunnel Hensing, Carl Bonander

**Affiliations:** 1 Health Economics and Policy, School of Public Health and Community Medicine, Institute of Medicine, University of Gothenburg, Gothenburg, Sweden; 2 Insurance Medicine, School of Public Health and Community Medicine, Institute of Medicine, University of Gothenburg, Gothenburg, Sweden

**Keywords:** Stroke, Spouse, Employment transitions, Labor force

## Abstract

**Background:**

The sudden occurrence of stroke often leads to impaired physical, emotional, and cognitive abilities. Many stroke survivors therefore require support from their family members. However, little is known about the effects of a stroke event on the spouses’ employment transition probabilities. The aim of this study was twofold 1) to investigate whether a first ever stroke has an effect on employment transition probabilities for employed and unemployed spouses and 2) to analyze whether heterogeneity with respect to age, gender, education and comorbidities influence the size of the effect.

**Method:**

Data for this population-based cohort study were extracted from Swedish national registries from 2005 to 2016. The national sample consisted of 1818 spouses of first ever stroke survivors during 2010 and 2011, and 7399 matched controls that were employed or unemployed during 5 years prior stroke onset. Effects of stroke on spousal employment transitions were analyzed using linear regression, stratified by employment status prior to stroke onset.

**Results:**

Employed spouses prior stroke onset reduced their employment by − 1.3 percentage points (95% CI, − 2.4, − 0.2). The data also indicated that employed spouses with lower age, comorbid conditions, and low educational attainment may be at even greater risk of transitioning to unemployment. On the other hand, stroke events appear to have limited impact on spouses that were unemployed prior to stroke onset.

**Conclusion:**

The risk of transitioning to unemployment appears to increase after stroke onset for spouses of stroke survivors, and disadvantaged groups may be at even greater risk. Thus, it is important for policy-makers to implement interventions to ensure that these groups of spouses have the possibilities to combine their caregiving role and remaining in the labor market.

## Introduction

Globally, in 2016, stroke was the second leading cause of death and third leading cause of disability [1]. One in four people over the age 25 will during their lifetime be diagnosed with stroke [1]. In Sweden, a country with 10 million inhabitants, approximately 20,000 patients are annually diagnosed with first ever stroke, whereof 20% are in working age [2]. The sudden occurrence of stroke often leads to impaired physical, emotional, and cognitive abilities [3]. The estimated prevalence of disability after stroke concerns about 40% of the stroke survivors in Sweden [4], which are often long-lasting in the middle-aged group [5]. Thus, spouses’ often provides long-term support which covers a wide range of activities [6] and can be perceived to bring positive aspects in life [7]. However, the informal support can also be demanding and have a negative impact on the spouses wellbeing, leisure activities, social functioning [8, 9], and health-related quality of life [10].

During the recent decades there has been a significant increase in the incidence of stroke survivors in working ages [11]. Many working-age stroke survivors perceive a reduction in their ability to work after the stroke onset [12]. Economic theory suggests that couples often share their resources and make joint decisions about their work [13, 14]. Nevertheless, previous studies have found little change in spouses’ employment after a partner’s health shock on average [15–19]. Possible explanations of these small changes could be that some spouses may increase their labor supply to add up for the lost income from employment by their partner, i.e. the “added worker effect” [14, 20], while other spouses may decrease their labor supply in order to care for their sick partner, i.e. the “caregiver effect” [21]. When assessing the average effect on spouses employment rates these two forces may cancel each other out when comparing the spouses’ employment to unexposed controls. Such cancellations, if they exist, can be disentangled by studying employment transitions separately for spouses that were employed and unemployed before stroke onset. The aim of this study was twofold 1) to investigate whether a first ever stroke has an effect on employment transition probabilities for employed and unemployed spouses and 2) to analyze whether heterogeneity with respect to age, gender, education and comorbidities influence the size of the effect.

## Method

### Data sources

This longitudinal population-based cohort study was conducted by linking Swedish registry data based on unique personal identity numbers available for each member of the Swedish population [22]. We used the following five national registries to construct our dataset. The *Swedish Stroke Registry* was used to identify stroke survivors with stroke onset during the years 2010 and 2011. The degree of coverage for this register is estimated to be 89% [2]. The *National Population Registry* was used to identify stroke survivors with a cohabitant spouse or partner and to identify the personal identity number of the spouse or partner. A spouse or partner was defined as the adult husband/wife/legal partner living at the same address as the patient at the year of the stroke onset. For brevity, we refer to this study group as spouses. This registry was also used to match the spouses to a reference cohort from the general population to be used as controls (see below). The *Longitudinal Integration Database for Health Insurance and Labor Market Studies* was used to obtain socioeconomic status (SES) data and employment status by year relative to stroke onset. The National Patient Registry was used to identify diagnosis-specific data on hospitalization and specialized outpatient care, coded according to the International Classification of Diseases (ICD-10) [23].

Statistics Sweden performed the linkage of the registries and the authors received de-identified data-files. This study was approved by the Regional Ethics Committee in Gothenburg, Sweden, with reference number 813–17.

### Study population

The study group consisted of cohabitant spouses of stroke survivors with first ever stroke onset during 2010 and 2011. We followed each individual in the sample over 11 years; 5 years prior to stroke onset (t = − 5, t = − 4, …, t = − 1), the year of onset (t = 0), and for five follow-up years (t = 1, t = 2 …, t = 5). To accommodate this design, our sample was restricted to spouses and matched controls aged ≤60 years at stroke onset to allow for at least 5 years of follow-up before they reach retirement age. To be included, the individuals also had to be either employed (full-time or part-time) or unemployed during the entire pre-stroke period (i.e., employed or unemployed at t = − 5, t = − 4, …, t = − 1).

**Fig. 1:**
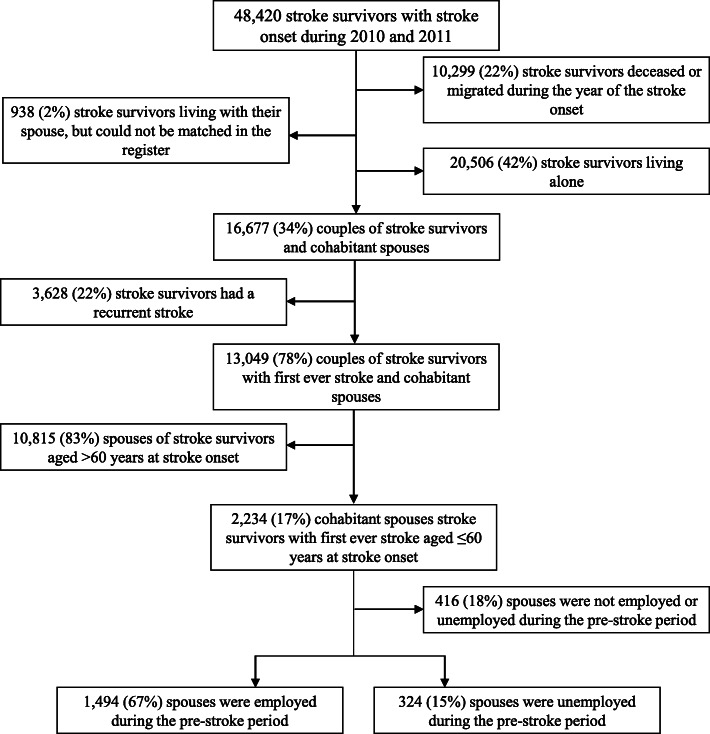
Flowchart of study population

The identification of stroke survivors’ cohabitant spouses could not be done for couples living in apartment blocks without shared custody of their children, and these persons were, therefore, not included in the study. This exclusion represents 2% of all survivors with stroke onset during 2010 and 2011 (Fig. 1).

The controls were selected from the general population via one-to-four matching to each spouse by age, gender, and place of residence. Each individual from the controls could only be matched to one spouse and none of the spouses could be matched as an individual in the controls. Statistics Sweden performed the matching procedure of both stroke survivors and their spouses, as well as matching of the spouse and the controls.

### Measurements

The following demographic variables were used as matching variables and were coded at the year of the stroke onset: *Age* (continuous variable); *Gender* female or male; *Geographical residence* based on six healthcare regions “Southern Sweden”, “Western Sweden”, “Southeastern Sweden”, “Capital area”, “Central Sweden” and “Northern Sweden”.

The following demographic variables were used as covariates and were coded at the year of the stroke onset: *Educational level* in three categories “less than high school” (i.e. 9 years of education), “high school” (i.e. 12 years of education), or “University or more” (i.e. more than 12 years of education).

To assess *medical comorbidities* among the spouses and individuals in the reference cohort the Charlson Comorbidity Index (CCI) was used [24]. The CCI was estimated by coding algorithms using ICD-10 codes [25] for hospitalizations and visits to specialist physicians during the study period.

### Statistical analyses

To examine effects on employment transition probabilities, we first stratified the data into two subcohorts as mentioned in the introduction: 1) spouses who were employed throughout the entire pre-stroke period (t = − 5, t = − 4, …, t = − 1) and their matched controls, and 2) those who were unemployed during the same period. We then conducted each of the following analyses separately for each subcohort.

Unadjusted difference in employment transition probabilities between spouses and controls (within each subcohort) were initially assessed using Pearson’s chi-squared test. We then used multiple ordinary least squares (OLS) regressions to estimate the effects of stroke on spousal employment probabilities. The outcome variable was defined as the employment probability averaged within each individual over the post-stroke period (t = 1, t = 2 …, t = 5). Our choice of model was motivated by the fact that our goal was to quantify effects on an additive scale, which are often considered more policy-relevant [26]. The sample size should be sufficiently large for normality assumptions to hold for the effect estimates. (We probed this assumption by running fractional logistic regression models as a sensitivity analysis [27], which lead to the same conclusions as those presented below.) The models were adjusted for confounding by means of matching and regression adjustment. The followed variables were matched on: age, gender, geographical residence (via the reference cohort design) and pre-stroke employment status (via the stratification into subcohorts detailed above). Educational attainment and medical comorbidities were adjusted for by including them as covariates in the model.

With this design, causal identification relies on the assumption that the potential outcomes under each exposure status *a* ∈ {0, 1}, *Y*_*i*_(*a*), are exchangeable (“as good as randomly assigned”) between spouses and controls conditional on observed covariates *X*_*i*_ and the vector of pre-event employment histories ${Y}_i^{T0}$, where superscript *T0* denotes that the outcomes are from the pre-event period. While it may be more common to model longitudinal data using panel data estimators [28], we note that individual fixed effects regressions or difference-in-differences estimation would be redundant in this case, because individuals within each subcohort are already perfectly matched with respect to pre-intervention outcomes. Thus, the individual-specific intercepts (fixed effects) would all be equal and pre-event group differences on the outcome would be zero. Conceptually, our approach therefore relates more closely to methods that match on pre-event outcomes, such as synthetic controls [29], except we rely on exact matching on ${Y}_i^{T0}$ rather than weighting.

In addition to the main analysis, we also performed five subgroup analyses using separate regression models to test for heterogeneity in the effect depending on age, gender, comorbidities, and educational attainment. All analyses were carried out in Stata (version 15.1, Stata, College Station TX, USA). The” regress” command was used to fit the OLS regression models, with interaction terms entered using the “##” subcommand to specify a full-factorial interaction model with main effects and interactions.

## Results

During 2010 and 2011, 48,420 stroke survivors with stroke onset were registered in the Swedish Stroke Registry. Of these stroke survivors, 22% deceased or migrated during the year of the stroke onset, 42% were living alone and 2% could not be matched to their spouse in the Swedish Stroke Registry. Thus, 16,677 (34%) of the stroke survivors had a registered cohabitant spouses in the Population Registry, whereof 13,049 (78%) where spouses of stroke survivors with first ever stroke during 2010 and 2011. Of these spouses, 2234 were aged ≤60 years or under at stroke onset, whereof 1494 (67%) were employed and 324 (15%) were unemployed during the pre-stroke period. The flowchart of the study population is presented in Fig. 1. The descriptive statistics for the study population are presented in Table 1.

**Table 1:** Descriptive statistics of study population

	Spouses *n* = 1818	Controls *n* = 7399
Age, mean (SD)	52.40 (6.85)	52.46 (6.72)
Females, n (%)	1361 (75)	5501 (74)
Education, n (%)		
Less than high school	349 (19)	1085 (14)
High school	859 (47)	3462 (47)
University or more	610 (34)	2852 (39)
Geographical residence, n (%)		
Southern Sweden	337 (19)	1373 (19)
Western Sweden	344 (19)	1394 (19)
Southeastern Sweden	192 (11)	791 (11)
Capital area	370 (20)	1539 (20)
Central Sweden	407 (22)	1610 (22)
Northern Sweden	168 (9)	692 (9)
Charlson Comorbidity Index (%)		
No comorbidities	1496 (82)	6472 (87)
Comorbidities (≥1)	322 (18)	927 (13)
Stroke-related outcomes of stroke surviving partner (%)		
Intracerebral hemorrhage	224 (12)	–
Cerebral Infarction	1556 (86)	–
Other^a^	24 (2)	–
Support by caregivers^b^	556 (31)	–
Support by social care^c^	468 (26)	–

^a^Other: Subarachnoid hemorrhage, Other and unspecified nontraumatic intracranial hemorrhage, Occlusion and stenosis of precerebral arteries, not resulting in cerebral infarction, Other cerebrovascular diseases, and Sequelae of cerebrovascular disease

^b^Reported by the stroke survivors in the Swedish Stroke Registry and refers to support by relatives, not only spouses

^c^Reported by the stroke survivors in the Swedish Stroke Registry and refers to home care, personal assistance, or living at nursing homes

Of the employed spouses during the pre-stroke period, 1249 (84%) spouses remained employed at 5 years post stroke onset, while 245 (16%) spouses transitioned to unemployment (Table 2). Of the unemployed spouses during the pre-stroke period, 269 (83%) spouses remained unemployed at 5 years post stroke onset, while 55 (17%) transitioned into employment (Table 2).

**Table 2:** Employment transitions for spouses of stroke survivors and matched controls

Employed at t = −5 to t = − 1	Employed at t = 5	Spouses of stroke survivors (%) *n* = 1818	Controls (%) *n* = 7399	*p*-value
Yes	**–**	1494 (82)	6463 (87)	
Yes	Yes	1249 (87)	5520 (88)	0.033
Yes	No	245 (13)	916 (12)	
No	**–**	324 (18)	963 (13)	
No	No	269 (83)	799 (83)	0.975
No	Yes	55 (17)	164 (17)	

**Fig. 2:**
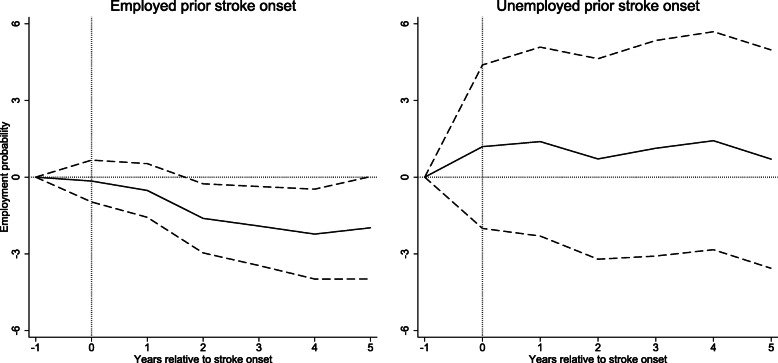
Estimated effects of stroke on spouses’ employment in percentage points for employed and unemployed spouses and control prior stroke onset. Solid lines represents spouses of stroke survivors and dashed lines represents the matched controls. Vertical lines represents the year of the stroke onset (t = 0). The models are adjusted for the following spousal variables: age, gender, educational level, geographical residence, and comorbidities

**Fig. 3:**
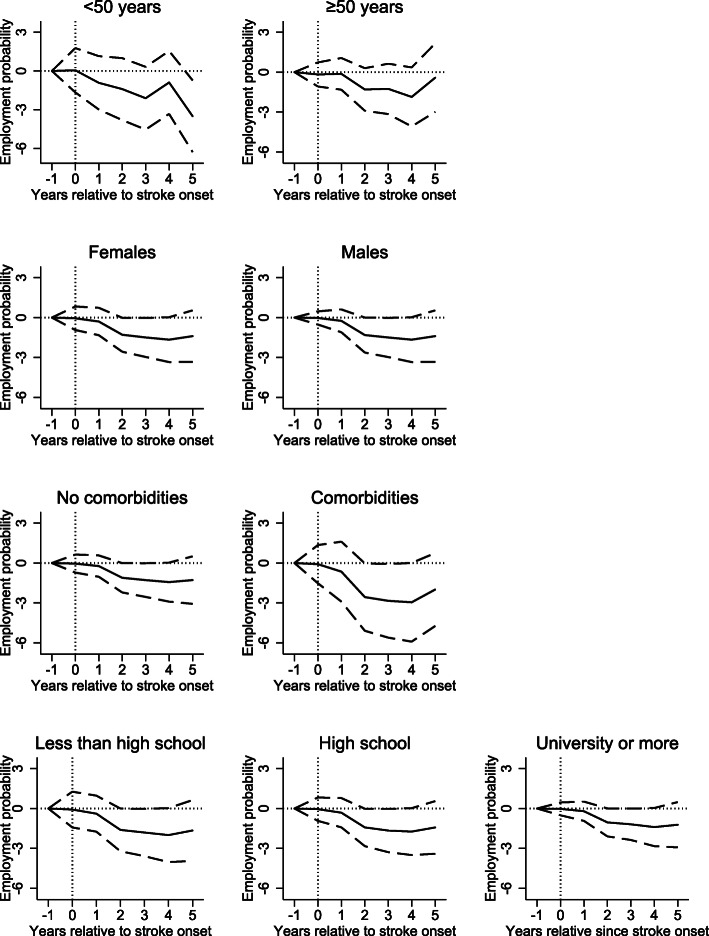
Estimated effects of stroke on spouses’ employment in percentage points for employed spouses and control prior stroke onset presented separately for subgroups. Solid lines represents spouses of stroke survivors and dashed lines represents the matched controls. Vertical lines represents the year of the stroke onset (t = 0). The models are adjusted for the following spousal variables: age, gender, educational level, geographical residence, and comorbidities

The full sample results indicated that stroke onset reduced employed spouses post-event employment probability by 1.3 percentage points (Table 3, also illustrated over time in Fig. 2). Figure 3 illustrates how heterogeneity with respect to age, gender, comorbidity and educational attainment influence the size of the effect over time. Females and males have similar trends in reduced post-event employment probability over time. However, spouses of younger age, with comorbidities and lower educational attainment transited more to unemployment over time compared to controls with comorbidity. However, we found no strong evidence of effect heterogeneity with respect to age, gender, comorbidities and educational attainment in this subcohort (Table 3).

**Table 3:** Full and subgroup analyses of employment transitions for spouses of stroke survivor and matched controls employed and unemployed prior to stroke onset (t = −5 to −1)

	Employed prior stroke onset		Unemployed prior stroke onset	
	Estimated effect on employment probabilities (Percentage points, 95% CI)	*p*-values for interactions	Estimated effect on employment probabilities (Percentage points, 95% CI)	*p*-values for interactions
Total sample	−1.3 (−2.4, −0.2)		1.4 (−2.1, 4.8)	
Age		0.644		0.637
<50y	−1.7 (−3.8, 0.5)		2.8 (−3.3, 0.9)	
≥50y	−1.1 (−2.4, 0.2)		1.1 (− 3.1, 5.2)	
Gender		0.990		0.586
Female	−1.3 (−2.6, −0.1)		3.9 (−5.1, 13.0)	
Male	−1.3 (−3.4, 0.8)		1.2 (−2.5, 4.9)	
Educational level		0.595		0.065
Less than high school	−2.6 (−5.5, 0.3)		5.5 (−0.1, 11.1)	
High school	−0.9 (−2.5, 0.7)		−3.2 (−8.5, 2.1)	
University or more	−1.2 (−3.0, 0.6)		4.2 (−3.1, 11.6)	
Comorbidities		0.055		0.192
No (CCI = 0)	−0.8 (−2.0, 0.4)		3.0 (−1.0, 6.9)	
Yes (CCI ≥ 1)	−3.9 (−6.8, −1.0)		−2.2 (−9.1, 4.5)	

The models are adjusted for the following spousal variables: age, gender, educational level, geographical residence, and comorbidities

For spouses unemployed prior stroke onset, the full sample results indicated no substantial effect on the average employment rate during the post-stroke period (Table 3), also illustrated in Fig. 2. We found no strong evidence of effect heterogeneity with respect to age, educational level and comorbidities either in this subcohort.

## Discussion

This nationwide population-based cohort study investigated whether there was an effect of stroke on their spouses’ employment transition. To avoid that the effect on spouses’ employment rates would be cancelled out by some spouses decreased and others increased their labor supply after the stroke onset of their partner, we investigated the employment transitions separately for spouses that were employed and unemployed during five before the stroke events. The findings in this study showed that spouses who were employed prior to stroke onset had a 1.3 percentage points lower employment probability after the event compared to controls, and that spouses of younger age, with comorbid condition and low educational attainment may be at even greater risk of transitioning to unemployment.

Previous studies have shown that many stroke survivors experience long-lasting reductions in their ability to work and earn income [12, 30, 31]. One of the most consistent predictors of stroke survivors return to work is stroke severity [32, 33]. Stroke survivors with higher SES are more likely to return to work after the stroke event [30, 31]. While economic theory suggests that couples often share their resources and make joint decisions about their work [13, 14], previous studies have found little change in spouses’ employment after a partner’s health shock on average [15–19]. Jeon et al. [19] found effect heterogeneity with respect to stroke severity and age, where spouses of stroke survivors with more severe stroke as well as younger spouses were more likely to transit to unemployment after the stroke events. Similar to these findings, our results indicated that younger spouses were more likely to transit to unemployment, compared to spouses older than 50 years. Possible explanations for this findings could be that a partner’s stroke event might imply greater lifestyle changes for younger spouses than for older employed spouses [34], since spouses in this younger age group often have responsibilities of both family and working life [35].

Furthermore, previous research has shown that there is spousal concordance concerning cardiovascular risk factors, such as smoking habits, sedentary life, overweight and high blood pressure [36], high alcohol consumption and a poor diet [37]. Recent meta-analyses have also confirmed high rates of spousal concordance for hypertension [38] and diabetes [39]. The spouses of stroke survivors observed in our sample had more comorbidities compared to the controls, which support these findings. Nonetheless, even when analyzing the subgroup of spouses and controls with comorbidities, the spouses with comorbidities were more likely to transit to unemployment to a larger extent compared to controls with comorbidities. This finding suggests that spouses with comorbidities may experience a greater burden of the caregiving role that might result in difficulties to maintain their working life. Jansen et al. [40] found in a study of self-reported family-work conflicts that accommodations of working hours were more common in those who reported a family-work conflict. It can be hypothesized that spouses with comorbidities already experience family – work conflicts and when hit by a stroke event in the family the balance might be difficult to withstand. If so, this could serve as a possible explanation for the effect heterogeneity found with respect to comorbidities in our data.

Garcia-Gomez et al. [17], found that the impact on spousal employment after hospitalization of a partner varied with income. Spouses in the richest household significantly reduced their employment after the health shock, whereas there was no difference in employment among the spouses in the poorest households. The authors argues that the spouses in the richest households may afford to reduce their employment or taking early retirement to spend more time with their partner, while spouses in the poorest household do not have this possibility to the same extent. In this current study, we found evidence suggesting that the effect size of stroke on spouses’ employment probability for employed spouses’ prior stroke to become larger over time for spouses with lower educational attainment. The main differences between these findings is that Garcia-Gomez et al. [17] investigated the impact on spousal employment after a partners health shock in a more general population, while in this current study we analyzed the same impact in a specific population of families of stroke survivors. Families of stroke survivors is overrepresented among individuals with low SES [41, 42] and are thus in a more vulnerable situation already before the stroke. Lower SES has been shown to negatively affect return to work for stroke survivors after stroke [30, 31], and our finding indicate that low educational attainment also affect transit to unemployment for employed spouses prior stroke onset. A possible explanation for this could be that women in groups with lower SES have weaker connection to the labor market [43]. The risk of unemployment is greater for individuals who are easier to replace in the labor market, such as individuals with short-term employment contracts, or working part-time hour by hour that is common in female-intensive occupations such as healthcare and elderly care. Given that the majority of working-age spouses of stroke survivors are women, it is important for policy-makers to implement intervention to improve the employment security to increase the possibilities of spouses in lower SES to remain in the labor market when also being informal caregivers.

### Strengths and limitations

The main strength of this study was the quality of data from national registries, including approximately 80% of all stroke survivors with stroke onset during 2010–2011, based on the coverage by the Swedish Stroke Registry. The choice of studying spouses of stroke survivors is also advantageous from a scientific perspective due to the sudden and unexpected onset of the disease, which gives a clear cut before and after situation. However, this study has a number of limitations that should be mentioned. First, since this study was based on national registry data there are limitations in the depth of the information including caregiving status, intensity of caregiving, and whether the spouses experienced psychological or physiological distress. Previous investigation in the Swedish setting has shown that the extent of informal support have an impact on employment and educational attainment [44]. Thus, further research is needed to investigate whether aspects, such as caregiving strain and hours of informal caregiving, have an impact on the labor market outcomes for spouses of stroke survivors. Second, the assessment of comorbidity is based on data for hospitalizations and visits to the specialists’ physicians, while healthcare utilization within primary care were not included in this study. Consequently, not all medical conditions are captured within the comorbidity measurements in this study, especially poor mental health such as depression, anxiety, stress and burnout syndrome. Further studies also including primary care utilization are necessary to have a broader picture of the spouses’ comorbidities. Third, since the economic crisis in 2008 and 2009, the employment rate in Sweden has increased every year. During the follow-up period of this study (from 2011 to 2016), the employment rate in Sweden increased from 65.4 to 67.1% in the working-age population (15 to 74 years) [45]. Nonetheless, our findings show that the employment rate for employed spouses of stroke survivors’ decreased by 1.3 percentage points during the same period. This might imply that the impact of stroke on spouses’ employment transition might have been even greater if the follow-up period had been during a period of rising unemployment.

## Conclusion

Stroke is usually a sudden and unexpected event where spouses often have to enter the caregiving role without any warning and preparation. In this study, we show that employed spouse’s prior stroke onset reduced their employment. Our results also indicates that lower age, comorbidity and lower educational attainment influence the size effects of stroke on spouses’ employment probability. These subgroups seems to be more affected by the caregiving role. Thus, it is important for policy-makers to implement interventions to ensure that these groups of spouses have the possibilities to combine their caregiving role and remaining in the labor market.

## Data Availability

The data that support the findings of this study can be made available to other researchers provided that ethical approval has been obtained from a Swedish regional ethics committee. The data cannot be made publicly available due to ethical restrictions. Please contact the corresponding author for further details.

## Abbreviations

CCI: Charlson comorbidity index
CI: Confidence interval
ICD-10: International Classification of Diseases
OLS: Ordinary least squares (OLS)
SES: Socioeconomic status
